# Correction: Tracking the neurodevelopmental trajectory of beta band oscillations with optically pumped magnetometer-based magnetoencephalography

**DOI:** 10.7554/eLife.103993

**Published:** 2024-10-03

**Authors:** Lukas Rier, Natalie Rhodes, Daisie O Pakenham, Elena Boto, Niall Holmes, Ryan M Hill, Gonzalo Reina Rivero, Vishal Shah, Cody Doyle, James Osborne, Richard W Bowtell, Margot Taylor, Matthew J Brookes

**Keywords:** Human

Rier L, Rhodes N, Pakenham DO, Boto E, Holmes N, Hill RM, Reina Rivero G, Shah V, Doyle C, Osborne J, Bowtell RW, Taylor M, Brookes MJ. 2024. Tracking the neurodevelopmental trajectory of beta band oscillations with optically pumped magnetometer-based magnetoencephalography. *eLife* **13**:RP94561. doi: 10.7554/eLife.94561.

Published 4 June 2024

We discovered that the two leftmost panels in Figure 4A were duplicates, i.e. the image under “Middle 9 Children” was duplicated under “Youngest 9 Children. This error occurred due to an incorrect file being loaded when combining outputs saved during data analysis into our figure creation software. The error only affected those panels in Figure 4A; the values stated in the caption and reported statistics in the main text are correct.

The corrected Figure 4 is shown here:

**Figure 1:**
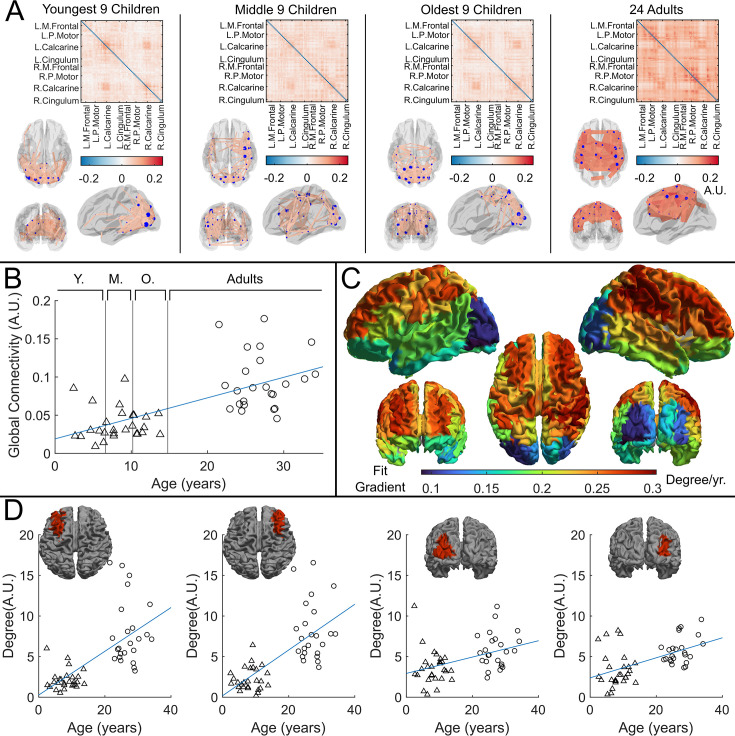


The originally published Figure 4 is shown for reference:

**Figure 2:**
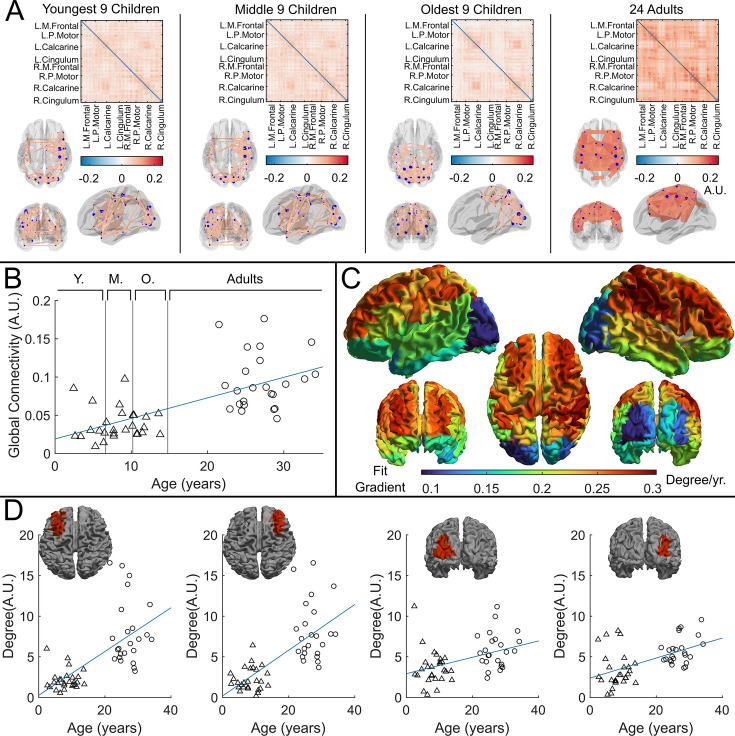


The article has been corrected accordingly.

